# Supplementary material for: DNA polymerase α/primase extraction from chromatin by VCP/p97 restricts ATR activation during unperturbed DNA replication
Source: Nat Commun. 2025 Jul 1;16:5706. doi: 10.1038/s41467-025-60077-w (PMC12219636; doi:10.1038/s41467-025-60077-w)
Supplement: Supplementary file 1 — Supplementary Information [file 41467_2025_60077_MOESM1_ESM.pdf]

SUPPLEMENTARY INFORMATION

DNA polymerase α/Primase extraction from chromatin by VCP/p97 restricts ATR activation during unperturbed DNA replication

Sara Rodríguez-Acebes[1#], Rodrigo Martín-Rufo[2#], Alicia Gómez-Moya[2], Scott B Churcher[2], Alejandro Fernández-Llorente[2], Guillermo de la Vega-Barranco[2], Alejandra Perona[2], Pilar Oroz[2], Elena Martín-Doncel[3], Luis Ignacio Toledo[3], Juan Méndez[1], Emilio Lecona[2*]

1 DNA Replication Group, Spanish National Cancer Research Centre (CNIO), Madrid 28029, Spain
2 Chromatin, Cancer and the Ubiquitin System lab, Centre for Molecular Biology Severo Ochoa (CBMSO, CSIC-UAM), Department of Genome Dynamics and Function, Madrid 28049, Spain
3 Center for Chromosome Stability, Institute for Cellular and Molecular Medicine, Faculty of Health and Medical Sciences, University of Copenhagen, Copenhagen 2200, Denmark

# equally contributed

*Correspondence: Emilio Lecona (elecona@cbm.csic.es),

Supplementary Figure 1

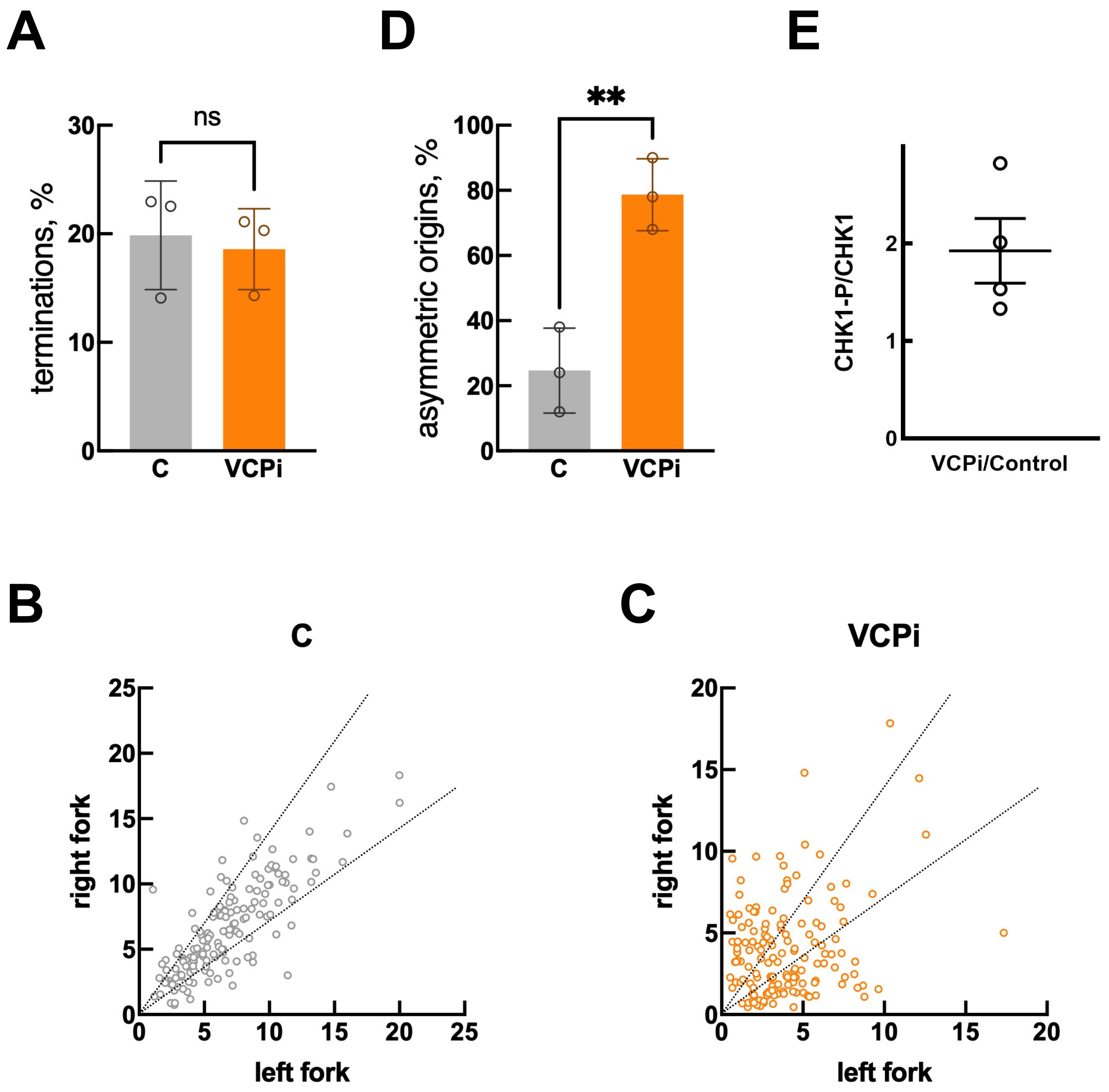

Supplementary Figure 1. Analysis of the effect of VCP/p97 inhibition on DNA replication termination and origin/fork symmetry. Related to Figure 1. Stretched DNA fiber analysis as in Figure 1C-H. (A) The percentage of terminated forks was quantified showing the mean of three independent experiments. Mean+/-SD, ns, non-significant in t-test. (B-C) Individual measures of the length of the right and left fork arising from a single origin was measured in control conditions (B) and after treatment with VCPi (C) corresponding to Figure 1H. The pool from three experiments is shown. Dotted lines represent a deviation of 40% from the bisectrice of the graph (ideal symmetric forks), as a reference for asymmetry. (D) The percentage of asymmetric origins was quantified showing the mean of three independent experiments. Mean+/-SD, **, p=0.0054 in t-test. (E) Densitometric quantification of the levels of CHK1-S345P normalized to the levels of total CHK1 in 4 independent experiments as shown in Figure 1I. Mean+/-SD.

Supplementary Figure 2

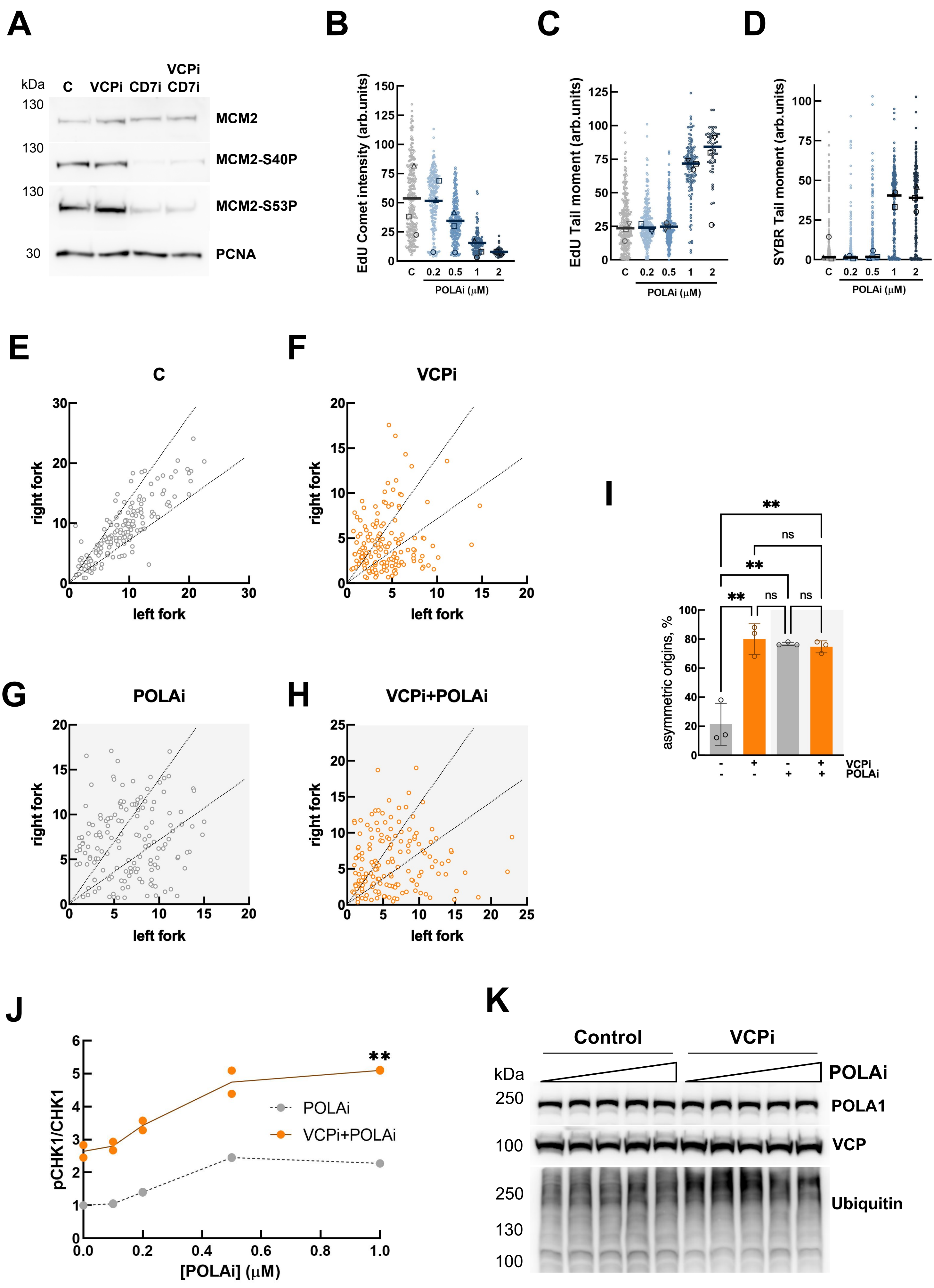

Supplementary Figure 2. Regulation of origin firing and fork progression by VCP/p97. Related to Figure 2.

(A) Western blot analysis in whole cell extracts of HCT116 cells treated as in Figure 2C-D. The levels of total and phosphorylated MCM2 (S40 and S53) and PCNA were measured with specific antibodies. The experiment was performed twice with similar results. (B-D) Comet assay on nascent DNA labelled with EdU. Cells were incubated with 30 µM EdU for 60 min, and EdU was conjugated to a fluorescent probe by a click reaction after alkaline comet assay. HCT116 cells were synchronized, released for 2.5 h and treated for 2 h with increasing concentrations of adarotene (POLAi) or DMSO as a control. The EdU tail moment (left), total DNA tail moment (center, SYBR) and total EdU intensity (right) were measured. Three independent experiments were combined and the median of individual experiments is shown with different symbols. (E-H) The length of the right and left fork arising from a single origin (from Figure 2H) was measured in control conditions (E) and after treatment with VCPi (F), POLAi (G) or a combination of both (H). The pool from three experiments is shown. Dotted lines represent a deviation of 40% from the bisectrice of the graph (ideal symmetric forks), as a reference for asymmetry. (I) The percentage of asymmetric origins was quantified showing the mean of three independent experiments. Mean+/-SD, **, $p<0.01$, ns, non-significant in one-way ANOVA with Tukey's test. (J) Densitometric quantification of the levels of phosphorylated CHK1 (S345) normalized to the levels of total CHK1 in 2 independent experiments as shown in Figure 2K. The levels were normalized to the ratio in control conditions. **, $p=0.0011$ in 2-way ANOVA with Sidak's post-test. (K) Western blot analysis as in Figure 2K showing that VCPi and POLAi treatment does not change the levels of POLA1, VCP or ubiquitylated proteins.

Supplementary Figure 3

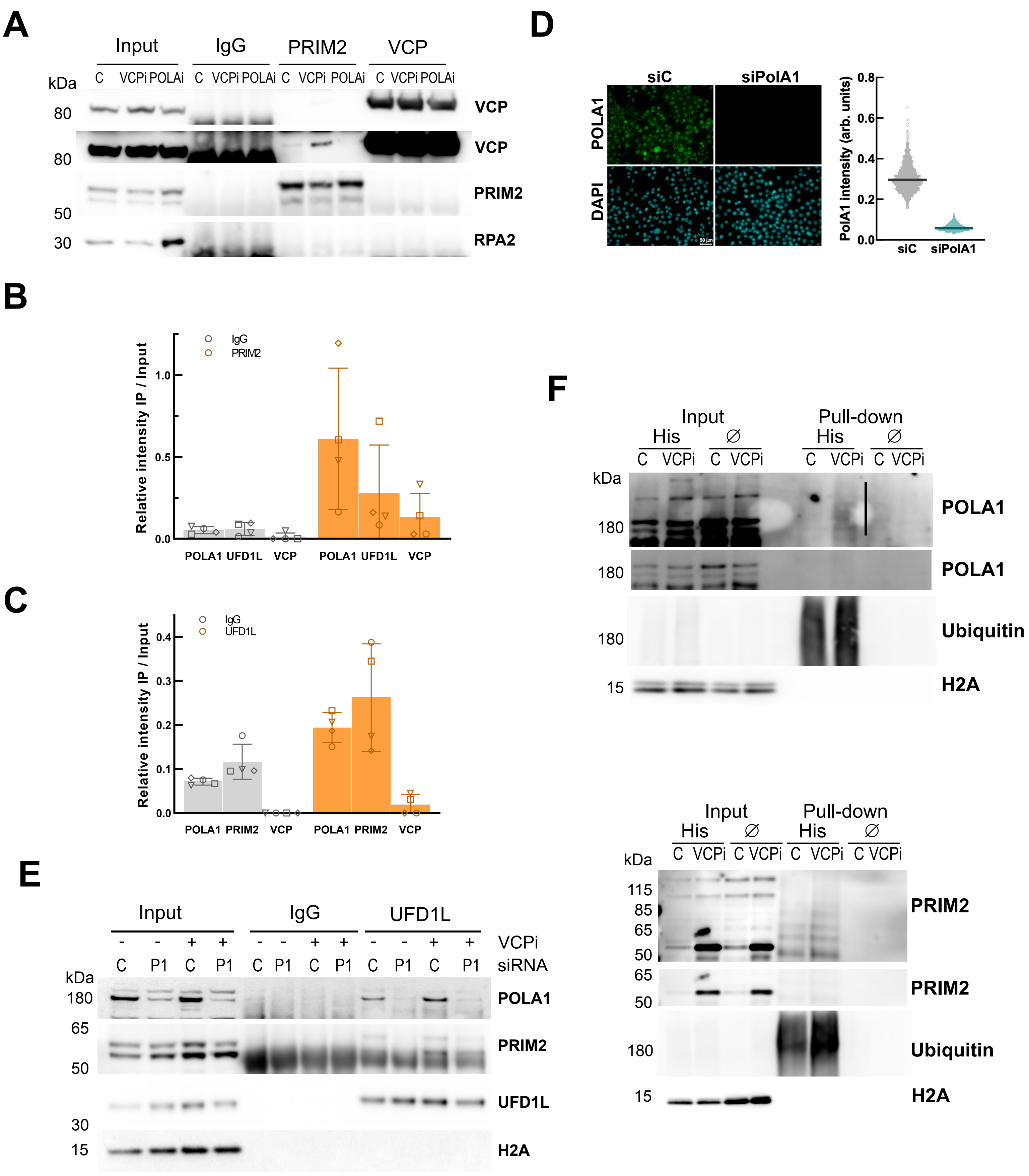

Supplementary Figure 3. POLA/PRIM is a substrate of VCP/p97. Related to Figure 3.

(A) Western blot analysis of the immunoprecipitation of PRIM2 and VCP/p97 from whole nuclear extracts obtained from HCT116 cells, synchronized, released for 2 h, treated for 3 h with 5 μM NMS873 (VCPi), 0.5 μM Adarotene (POLAi) or DMSO as a control (C). 2% of the input is shown together with the pull-down using a non-specific IgG as a negative control. A saturated exposure is included for VCP/p97. (B-C) Quantification of the immunoprecipitation experiments in Figure 3A-B. The amount of the indicated proteins in the pull-down of PRIM2 (B) and UFD1L (C) was normalized versus the input fraction. The levels in the control using a non-specific IgG normalized versus the input are shown. 4 independent experiments were used for the quantification. (D) Immunofluorescence of HCT116 cells transfected with an siRNA against POLA1 (siPolA1) or a non-specific siRNA as a control (siC). Nuclei were stained with DAPI. The quantification of the experiment is shown in the right. (E) Western blot analysis of the immunoprecipitation of UFD1L from whole nuclear extracts obtained from HCT116 cells transfected with a control siRNA (C) or an siRNA against POLA1 (P1), synchronized, released for 2 h and treated for 3 h with 5 μM NMS873 (VCPi) or DMSO as a control. 2% of the input material is shown together with the pull-down using a non-specific IgG as a negative control. The levels of POLA1, PRIM2 and UFD1L were analyzed with specific antibodies; histone H2A was used as control. (F) Western blot analysis of the immunoprecipitation of ubiquitylated proteins from whole nuclear extracts obtained from HCT116 cells transfected to express His-Ubiquitin (His), synchronized, released for 2 h and treated for 2 h with 5 μM NMS873 (VCPi) or DMSO as a control (marked as C). Mock transfected cells (ϕ) were used as control. Ubiquitylated proteins were purified and the presence of POLA1, PRIM2, Ubiquitin and histone H2A was analyzed with specific antibodies. A more exposed image for POLA1 and PRIM2 shows the smear for the ubiquitylated protein, indicated with a black bar for POLA1. 1% of the input is shown.

Supplementary Figure 4

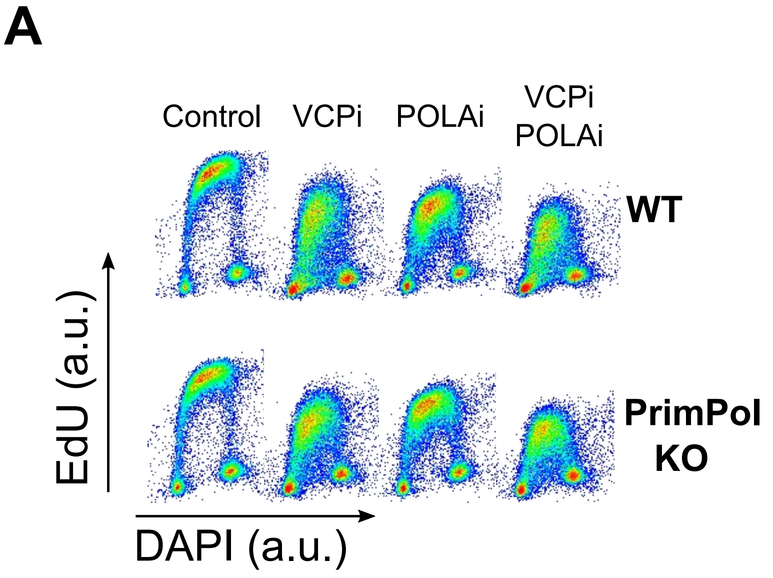

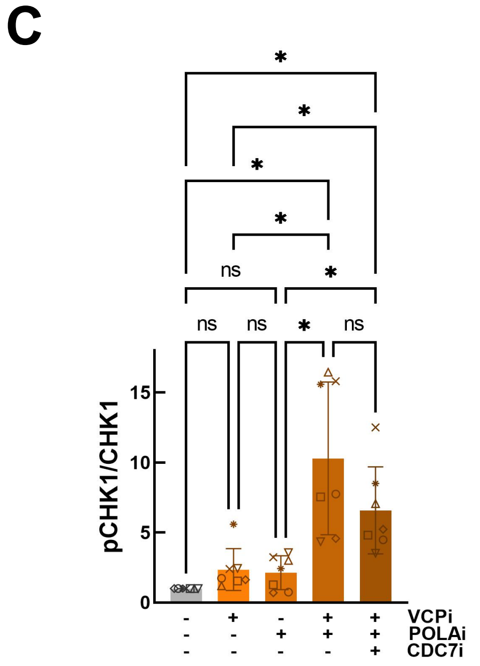

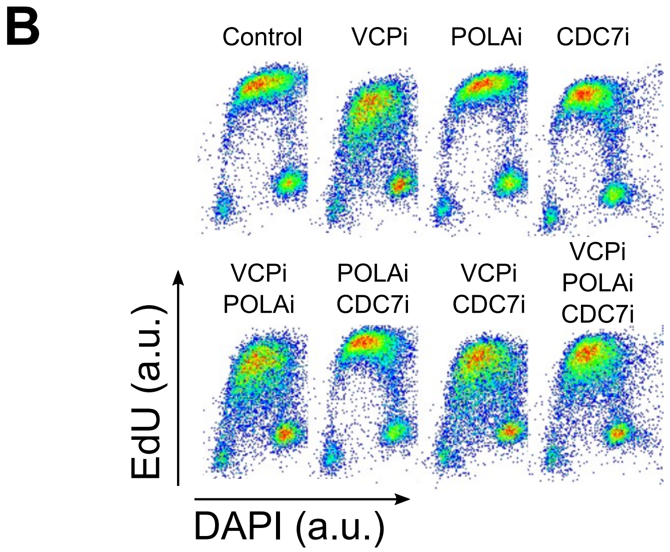

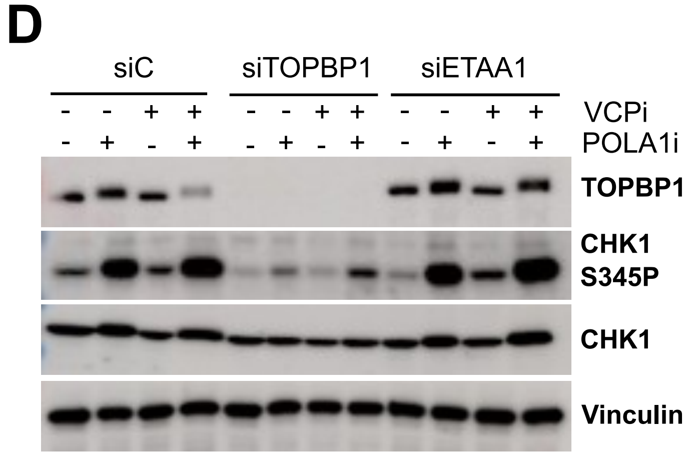

Supplementary Figure 4. The control of the replication stress response by VCP/p97 is related to origin firing. Related to Figure 3.

(A) Flow cytometry analysis of DNA content (DAPI) and DNA replication (EdU) in HCT116 cells wild-type and PRIMPOL knockout, synchronized, released for 2 h and treated with DMSO (marked as C), 5 μM NMS873 (VCPi), 0.5 μM adarotene (POLAi) or a combination of both (VCPi-POLAi) for 2 h. The experiment was repeated twice and one representative experiment is shown. (B) Flow cytometry analysis of DNA content (DAPI) and DNA replication (EdU) in HCT116 cells, synchronized, released for 2 h and treated with DMSO (marked as C), 5 μM NMS873 (VCPi), 0.5 μM adarotene (POLAi), 20 μM XL413 (CDC7i) or the indicated combinations for 2 h. The experiment was repeated three times and one representative experiment is shown. (C) Densitometric quantification of the levels of CHK1-S345P normalized to the levels of total CHK1 in 7 independent experiments as shown in Figure 3F. The levels were normalized to the ratio in control conditions. Mean+/-SD, *, $p<0.05$, ns, non-significant in one-way ANOVA with Holm-Sidak's test. (D) U2OS cells were transfected with a non-specific siRNA or siRNA pools directed against TOPBP1 or ETAA1. 72 h after the transfection the cells were treated with 10 μM NMS873 (VCPi), 0.5 μM adarotene (POLAi) or a combination of both using DMSO as a control. Western blot analysis of whole cell extracts with antibodies against TOPBP1, total and phosphorylated CHK1 (S345) using vinculin as a control. The experiment was performed twice with similar results.

Supplementary Figure 5

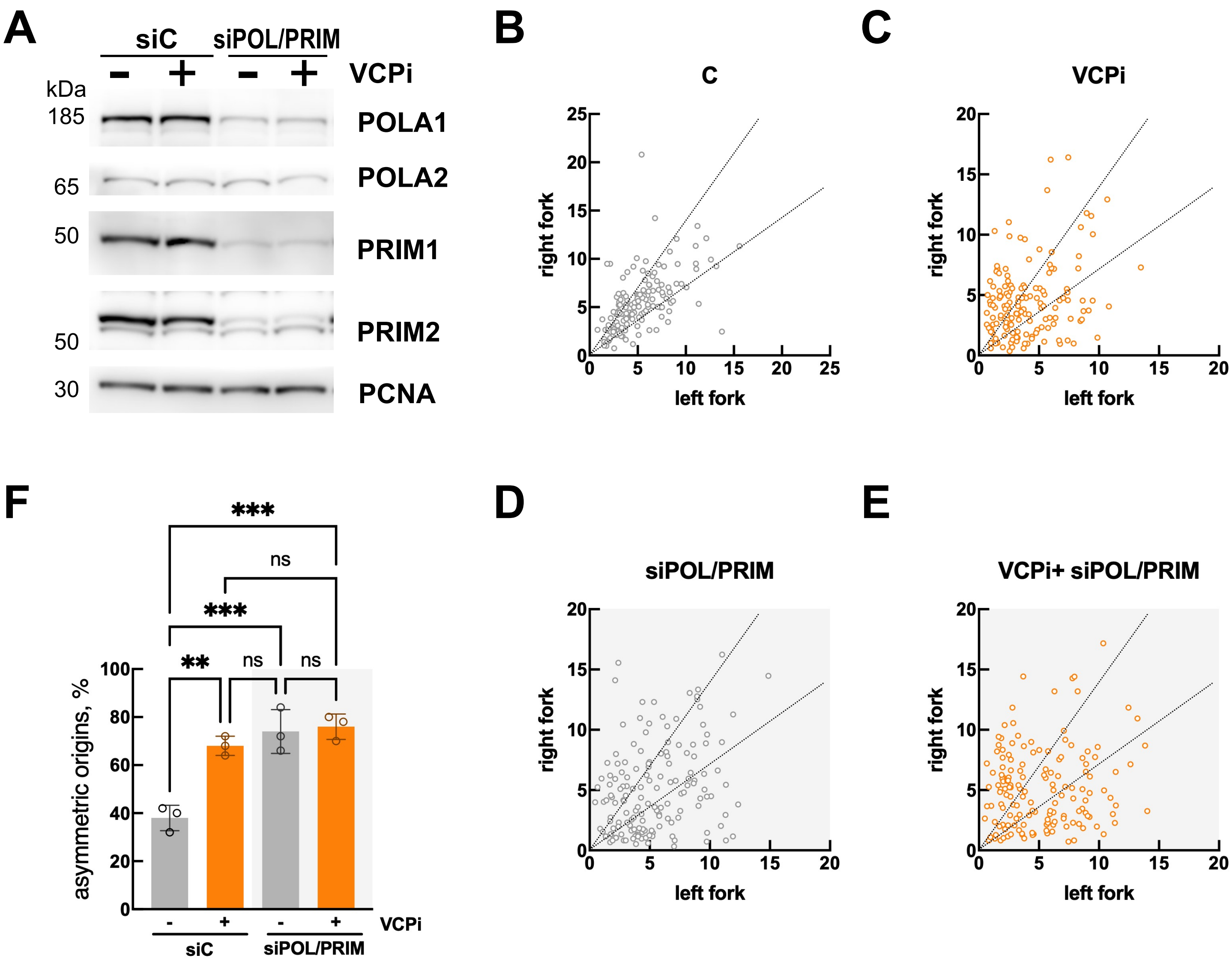

Supplementary Figure 5. Effect of the depletion of POLA/PRIM on DNA replication dynamics. Related to Figure 4.

(A) Western Blot analysis of the levels of POLA1, POLA2, PRIM1 and PRIM2 in HCT116 cells transfected with a control siRNA or a combination of 4 individual siRNA against POLA1, POLA2, PRIM1 and PRIM2, and treated with DMSO or with 5 μM NMS873 (VCPi), as in Figure 4. The experiments were repeated five times and a representative result is shown. (B-F) Stretched DNA fiber analysis as in Figure 4E-F. (B-E) Individual measures of the length of the right and left fork arising from a single origin was measured in cells transfected with a control siRNA (B), depleted of POLA/PRIM (C), after treatment with VCPi in control transfected cells (D) and in POLA/PRIM depleted cells (E) corresponding to Figure 4E. The pool from three experiments is shown. Dotted lines represent a deviation of 40% from the bisectrice of the graph (ideal symmetric forks), as a reference for asymmetry. (F) The percentage of asymmetric origins was quantified showing the mean of three independent experiments. Mean+/-SD, **, $p<0.01$, ***, $p<0.001$, ns, non-significant in one-way ANOVA with Fisher's test.

Supplementary Figure 6

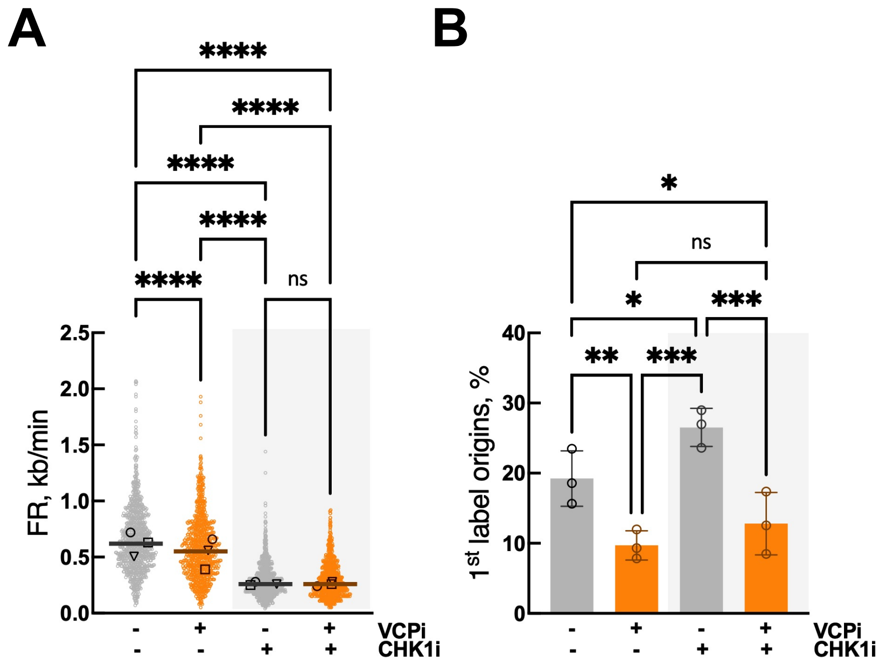

Supplementary Figure 6. Effect of VCPi and CHK1i in the dynamics of DNA replication. Related to Figure 5.

(A-B) Stretched DNA fiber analysis in HCT116 cells, synchronized with a single thymidine block, released for 2 h and treated for 2 h with 5 µM NMS873 (VCPi), 2.5 µM LY2603618 (CHK1i), a combination of both or DMSO as a control. Cells were sequentially incubated with CldU and IdU for 20 min. The fork rate (FR) (A) and percentage of origins fired during the first labelling time (1st label origins) (B) were measured and quantified after the different treatments. All the experiments were repeated three times and the pool of the experiments (FR, bars represent the median of the pooled data) or the average (1st label origins, mean+/-SD) is shown. The median of individual experiments is noted with different symbols both in FR and 1st label origins. *, $p<0.05$, **, $p<0.01$, ***, $p<0.001$, ****, $p<0.0001$, ns, non-significant in Kruskal-Wallis with Dunn's post-test (A) or one-way ANOVA with Tukey's test (B).

Supplementary Figure 7

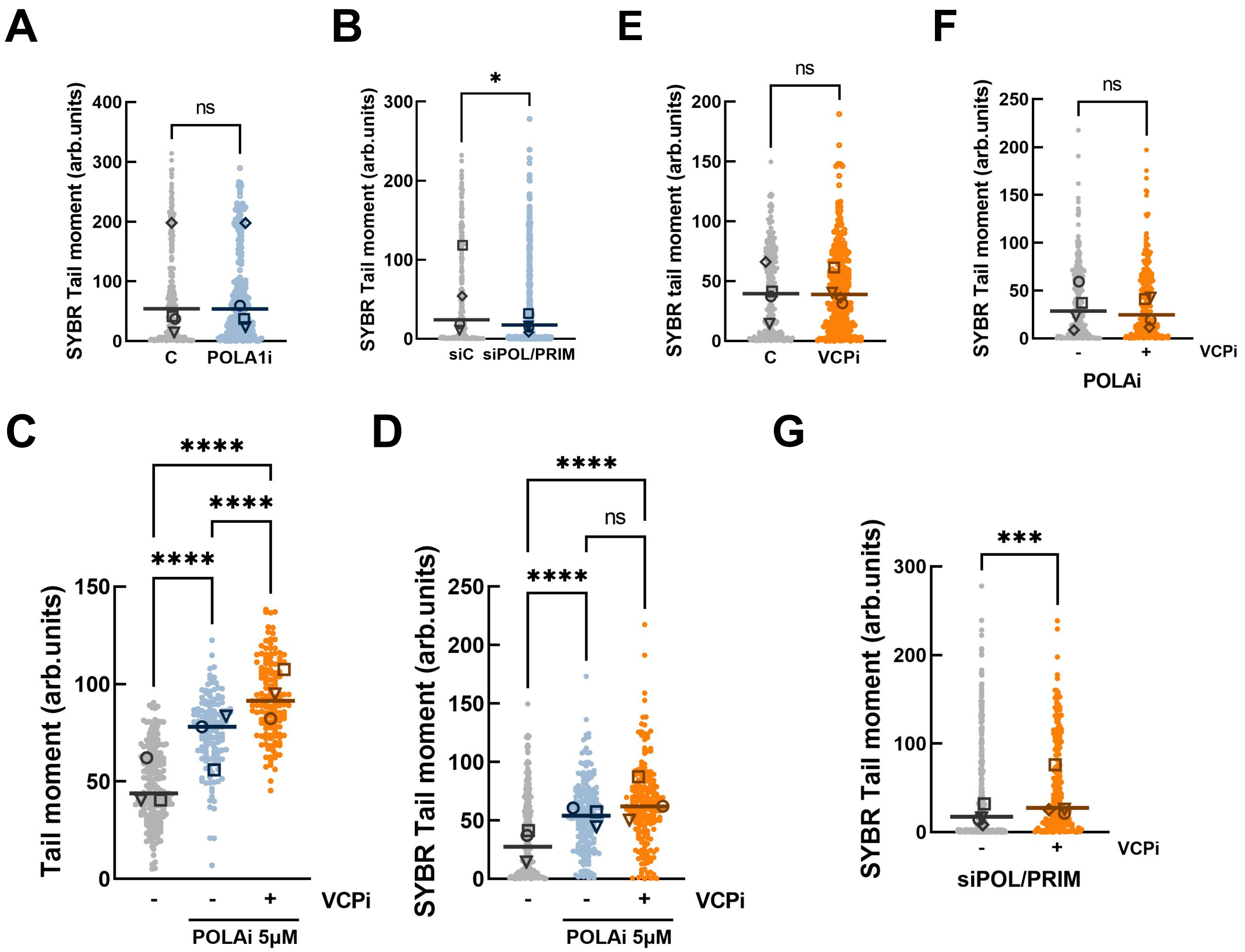

Supplementary Figure 7. Effect of VCPi in the generation of Okazaki fragments. Related to Figure 6.

Comet assay marking nascent DNA with EdU and total DNA with SYBR gold. Cells were incubated with 30 μM EdU for 60 min and EdU was conjugated to a fluorescent probe after alkaline comet assay. Total DNA was labeled with SYBR gold. (A-B) Tail moment of total DNA marked with SYBR gold from individual comets from four independent experiments corresponding to the treatment with 0.5 μM adarotene (POLAi) in Figure 6A-B (A) and to the depletion of POLA/PRIM in Figure 6C-D (B). (C-D) HCT116 cells were synchronized, released for 2.5 h and treated for 2 h with 5 μM adarotene (POLAi) or DMSO as a control. Tail moment from individual comets from three experiments is shown for EdU (C) and total DNA (SYBR, D). (E-G) Tail moment of total DNA marked with SYBR gold from individual comets from four independent experiments corresponding to the treatment with 5 μM NMS873 (VCPi) in Figure 6E-F (E), to the combined treatment with 0.5 μM adarotene (POLAi) and 5 μM NMS873 (VCPi) in Figure 6G-H (F), and to the treatment with 5 μM NMS873 (VCPi) in POLA/PRIM depleted cells in Figure 6I-J (G). In all cases, the individual medians of the independent experiments are noted with different symbols. Dot plots show pooled data of at least 4 experiments in A, B, E, F, G; or 3 experiments in C and D. Bars represent the median of pooled data. Individual medians of independent experiments are notes with different symbols. *, $p<0.05$; ***, $p<0.001$; ****, $p<0.0001$; ns, non-significant, in Mann-Whitney test (A, B, E, F, G) or Kruskal-Wallis followed by Dunns multiple comparisons test (C, D).
