# Supplementary material for: DNA polymerase α/primase extraction from chromatin by VCP/p97 restricts ATR activation during unperturbed DNA replication
Source: Nat Commun. 2025 Jul 1;16:5706. doi: 10.1038/s41467-025-60077-w (PMC12219636; doi:10.1038/s41467-025-60077-w)
Supplement: Supplementary file 2 — Reporting Summary [file 41467_2025_60077_MOESM2_ESM.pdf]

nature portfolio

Corresponding author(s): Emilio Lecona

Last updated by author(s): Apr 22, 2025

Reporting Summary

Nature Portfolio wishes to improve the reproducibility of the work that we publish. This form provides structure for consistency and transparency in reporting. For further information on Nature Portfolio policies, see our Editorial Policies and the Editorial Policy Checklist.

Statistics

For all statistical analyses, confirm that the following items are present in the figure legend, table legend, main text, or Methods section.

n/a	Confirmed	
☐	☒	The exact sample size (*n*) for each experimental group/condition, given as a discrete number and unit of measurement
☐	☒	A statement on whether measurements were taken from distinct samples or whether the same sample was measured repeatedly
☐	☒	The statistical test(s) used AND whether they are one- or two-sided<br>*Only common tests should be described solely by name; describe more complex techniques in the Methods section.*
☒	☐	A description of all covariates tested
☒	☐	A description of any assumptions or corrections, such as tests of normality and adjustment for multiple comparisons
☐	☒	A full description of the statistical parameters including central tendency (e.g. means) or other basic estimates (e.g. regression coefficient) AND variation (e.g. standard deviation) or associated estimates of uncertainty (e.g. confidence intervals)
☒	☐	For null hypothesis testing, the test statistic (e.g. *F*, *t*, *r*) with confidence intervals, effect sizes, degrees of freedom and *P* value noted<br>*Give P values as exact values whenever suitable.*
☒	☐	For Bayesian analysis, information on the choice of priors and Markov chain Monte Carlo settings
☒	☐	For hierarchical and complex designs, identification of the appropriate level for tests and full reporting of outcomes
☒	☐	Estimates of effect sizes (e.g. Cohen's *d*, Pearson's *r*), indicating how they were calculated

Our web collection on statistics for biologists contains articles on many of the points above.

Software and code

Policy information about availability of computer code

Data collection	N/A
Data analysis	N/A

For manuscripts utilizing custom algorithms or software that are central to the research but not yet described in published literature, software must be made available to editors and reviewers. We strongly encourage code deposition in a community repository (e.g. GitHub). See the Nature Portfolio guidelines for submitting code & software for further information.

Data

Policy information about availability of data

All manuscripts must include a data availability statement. This statement should provide the following information, where applicable:

- Accession codes, unique identifiers, or web links for publicly available datasets
- A description of any restrictions on data availability
- For clinical datasets or third party data, please ensure that the statement adheres to our policy

The raw data generated in this study are provided in the Source Data file.

Research involving human participants, their data, or biological material

Policy information about studies with human participants or human data. See also policy information about sex, gender (identity/presentation), and sexual orientation and race, ethnicity and racism.

Reporting on sex and gender	N/A
Reporting on race, ethnicity, or other socially relevant groupings	N/A
Population characteristics	N/A
Recruitment	N/A
Ethics oversight	N/A

Note that full information on the approval of the study protocol must also be provided in the manuscript.

Field-specific reporting

Please select the one below that is the best fit for your research. If you are not sure, read the appropriate sections before making your selection.

☒ Life sciences ☐ Behavioural & social sciences ☐ Ecological, evolutionary & environmental sciences

For a reference copy of the document with all sections, see nature.com/documents/nr-reporting-summary-flat.pdf

Life sciences study design

All studies must disclose on these points even when the disclosure is negative.

Sample size	N/A
Data exclusions	N/A
Replication	All experiments were repeated at least three times with similar results
Randomization	N/A
Blinding	N/A

Reporting for specific materials, systems and methods

We require information from authors about some types of materials, experimental systems and methods used in many studies. Here, indicate whether each material, system or method listed is relevant to your study. If you are not sure if a list item applies to your research, read the appropriate section before selecting a response.

Materials & experimental systems

n/a	Involved in the study	
☐	☒	Antibodies
☐	☒	Eukaryotic cell lines
☒	☐	Palaeontology and archaeology
☒	☐	Animals and other organisms
☒	☐	Clinical data
☒	☐	Dual use research of concern
☒	☐	Plants

Methods

n/a	Involved in the study	
☒	☐	ChIP-seq
☐	☒	Flow cytometry
☒	☐	MRI-based neuroimaging

Antibodies

Antibodies used	The antibodies against USP7 (Bethyl A300-033A), VCP (Bethyl A300-589A and Santa Cruz Biotechnologies sc-57492), SUMO2/3 (MBL, M114-3 and University of Iowa, clone 8A2), PCNA (Santa Cruz, sc-56), Chk1 (Novocastra and Cell Signalling #2360), Chk1-S345P (Cell Signaling #2348), RPA2 (Abcam ab2175), RPA2-S4/S8P (Bethyl, A300-245A), H2A (Cell Signaling #3636), γH2AX (Millipore, 05-636), Ubiquitin (Cell Signaling #3933), MCM2 (custom made, Juan Méndez86), MCM2-S40P (Abcam, ab133243), MCM2-S53P (Abcam,

	ab70367), POLA1 (Abcam, ab31777), PRIM2 (Invitrogen PA5-88189), TOPBP1 (Bethyl, A300-111), Vinculin (Sigma-Aldrich V9264), UFD1L (Abcam, ab155003), Histidine (BioRad MCA1396GA) were used for Western Blot and immunofluorescence. VCP (Abcam, ab11433 and Santa Cruz Biotechnologies sc-57492), PRIM2 (Invitrogen PA5-88189) and POLA1 (Abcam, ab31777) antibodies were used for immunoprecipitation.
Validation	All Bethyl antibodies are validated by WB and IP. The antibodies for SUMO2/3, PCNA, Chk1, Chk1-S345P, RPA2, RPA2-S4/8P, γH2AX, H2A, Ubiquitin and VCP have been previously validated in several publications. The antibodies against POLA1, PRIM2 and UFD1L were validated by silencing of the target proteins, WB and IP. The antibody against POLA1 was validated for IF by silencing of the target proteins. Antibodies against vinculin have been widely characterized. Antibody against Histidine has been validated by BioRad.

Eukaryotic cell lines

Policy information about cell lines and Sex and Gender in Research

Cell line source(s)	HCT116 (ATCC, CCL-247)
Authentication	None
Mycoplasma contamination	All cell lines were routinely tested for Mycoplasma contamination.
Commonly misidentified lines (See ICLAC register)	N/A

Plants

Seed stocks	N/A
Novel plant genotypes	N/A
Authentication	N/A

Flow Cytometry

Plots

Confirm that:

☒ The axis labels state the marker and fluorochrome used (e.g. CD4-FITC).

☐ The axis scales are clearly visible. Include numbers along axes only for bottom left plot of group (a 'group' is an analysis of identical markers).

☒ All plots are contour plots with outliers or pseudocolor plots.

☐ A numerical value for number of cells or percentage (with statistics) is provided.

Methodology

Sample preparation	For the analysis of the cell cycle, cells were incubated with 20 µM EdU for 30 minutes. Then, cells were trypsin-digested, washed with cold PBS once and fixed in 4% PFA/PBS for 15’ at room temperature. After permeabilization with 0.25 % Triton/PBS for 20’ at room temperature, the EdU was labelled by a Click reaction with a fluorescent azide in the presence of 10 mM Sodium Ascorbate, 2 mM $CuSO_4$ in PBS for 30’ at room temperature. Then, the DNA was stained with DAPI 0.5 µg/ml in the presence of 0.25 mg/ml RNAse A.
Instrument	BD LSRFortessa or FACSCanto II
Software	FACS Diva and FlowJo (v10)
Cell population abundance	N/A
Gating strategy	Cells were gated using FSC/SSC, then single cells were gated using FSC height and amplitude.

☐ Tick this box to confirm that a figure exemplifying the gating strategy is provided in the Supplementary Information.
